# Evaluating the safety and effectiveness of a nurse-led outpatient virtual IV vancomycin monitoring clinic: a retrospective cohort study

**DOI:** 10.1093/jacamr/dlaa113

**Published:** 2021-01-18

**Authors:** Kate S Grattan, Mohamed Mohamed Ali, Seyed M Hosseini-Moghaddam, Hayley J I Gilmour, Gregory P Crunican, Erica Hua, Kelly A Muhsin, Rochelle Johnstone, Lise C Bondy, Megan K Devlin, Sarah Shalhoub, Sameer Elsayed, Michael S Silverman

**Affiliations:** St Joseph’s Health Care, London, Ontario, Canada; Faculty of Nursing, Western University, London, Ontario, Canada; St Joseph’s Health Care, London, Ontario, Canada; St Joseph’s Health Care, London, Ontario, Canada; Division of Infectious Diseases, Department of Medicine, University Health Network, University of Toronto, Toronto, Ontario, Canada; St Joseph’s Health Care, London, Ontario, Canada; St Joseph’s Health Care, London, Ontario, Canada; St Joseph’s Health Care, London, Ontario, Canada; St Joseph’s Health Care, London, Ontario, Canada; London Health Sciences Centre, London, Ontario, Canada; St Joseph’s Health Care, London, Ontario, Canada; Division of Infectious Diseases, Western University, London, Ontario, Canada; St Joseph’s Health Care, London, Ontario, Canada; Division of Infectious Diseases, Western University, London, Ontario, Canada; St Joseph’s Health Care, London, Ontario, Canada; Division of Infectious Diseases, Western University, London, Ontario, Canada; St Joseph’s Health Care, London, Ontario, Canada; London Health Sciences Centre, London, Ontario, Canada; Department of Pathology & Laboratory Medicine, Western University, London, Ontario, Canada; St Joseph’s Health Care, London, Ontario, Canada; Division of Infectious Diseases, Western University, London, Ontario, Canada

## Abstract

**Background:**

Outpatient parenteral antimicrobial therapy (OPAT) with vancomycin is a common treatment modality for certain Gram-positive infections. Data regarding the safety of various models of delivery are limited.

**Objectives:**

To review outcomes of a nurse-led OPAT vancomycin monitoring service.

**Methods:**

This was a retrospective cohort study of consecutive patients referred to a nurse-led OPAT vancomycin clinic from December 2015 to March 2018. Patients were administered IV vancomycin in the home with active laboratory monitoring of vancomycin trough levels, renal function and complete blood count using an integrated electronic database linked with community laboratories (virtual vancomycin clinic, VVC). Monitoring was coordinated by nurses with physician approval of recommended dosing changes. Data were extracted from the electronic medical record. Demographics; clinical indication; microbial aetiology; culture source; antimicrobial regimen(s); serum creatinine and vancomycin trough values; initiation, discharge and completion dates; hospitalizations; adverse events; and outcomes were all evaluated.

**Results:**

Two hundred and seventy-five patients underwent a total of 301 courses of OPAT with vancomycin; 285 courses were completed. The rate of treatment discontinuation due to adverse effects was 33/301 (11.0%), with 15/33 (45.5%) being due to renal adverse effects (15/301 [5.0%] of episodes). Two of 15 (18.2%) patients developed stage 2 acute kidney injury (AKI), and no patients had stage 3 AKI or required haemodialysis. Nine of 301 (3.0%) required readmission for treatment failure. Nursing costs associated with monitoring were $63.93 CAD/patient ($48.43 USD).

**Conclusions:**

A nurse-led VVC was a safe, effective and inexpensive modality for administering outpatient vancomycin.

## Introduction

Outpatient parenteral antimicrobial therapy (OPAT) programmes have become increasingly popular since their introduction in 1974, as they have been shown to reduce costs, length of stay and acquisition of nosocomial infections and to improve patient satisfaction.^1^ However, adverse drug-related events (ADEs) have been a major concern in both inpatient^2^ and outpatient^3^ IV antimicrobial therapy programmes. The largest study to date of OPAT showed that clinically significant ADEs (i.e. hospital admission, change in antimicrobial therapy, antimicrobial discontinuation or development of *Clostridioides difficile* infection) were very common.^3^ Clinically significant ADEs occurred in 49/339 (14.5%) patients at an incidence of 2.24/1000 patient days. Independent risk factors for ADEs were female gender and receipt of vancomycin or daptomycin. Of 89 patients treated with vancomycin, 19 (21.3%) had a clinically important ADE, most commonly nephrotoxicity (11/89, 12.4%).^3^ Notably, higher vancomycin trough levels have been independently shown to correlate with a significant rise in nephrotoxicity risk.^4^

Outpatient management of invasive MRSA and methicillin-resistant coagulase-negative staphylococcal infections still depends heavily on IV therapies. Oral therapy for these infections is still evolving in clinical practice, with many clinicians remaining hesitant to utilize them.^1^ Long-acting lipoglycopeptides (e.g. oritavancin, dalbavancin) have recently been released in the USA as ‘lineless alternatives’, but data regarding their utility in invasive infections are still limited, and they are not generally available in Canada.^5^ Quality indicators for *Staphylococcus aureus* bacteraemia still recommend prolonged IV therapy,^2^ while cost restrictions sharply limit local availability of outpatient daptomycin. Therefore, in Canada, vancomycin remains the agent of choice for the treatment of invasive MRSA and several other invasive Gram-positive bacterial infections. 

Therapeutic drug monitoring of vancomycin in the outpatient setting remains quite challenging. Proper monitoring may not occur for a variety of reasons, including immobility, non-adherence, geographic isolation, lack of patient or provider knowledge, poor transitions of care, and laboratory constraints.^3^ These barriers can lead to default of monitoring, and the risks of inadequate therapy or increased toxicity. Inpatient vancomycin monitoring relies on physician or pharmacist supervision, while in outpatient settings these resources may be either unavailable or prohibitively expensive. To optimize the safety of vancomycin OPAT therapy within sustainable constraints, we developed a nurse-led virtual vancomycin clinic (VVC). We conducted a retrospective cohort review of patients referred to the VVC who had been initiated on parenteral vancomycin and required outpatient vancomycin therapy at home. The primary objective of this study is to review the clinical outcomes of these patients to determine the safety and efficacy of a nurse-led VVC.

## Patients and methods

### Cohort

In this retrospective cohort study, we included all patients at least 18 years of age referred to the VVC for home parenteral vancomycin by any one of the three tertiary-care academic medical centres in London, Ontario, between December 2015 and February 2018. Eligible patients were discharged from inpatient services or outpatient clinics and required parenteral vancomycin therapy through central or peripheral venous access for indications validated by infectious diseases (ID) consultants. Patients were ineligible if they were on haemodialysis, did not speak English or were unable to verbally consent. Eligible patients were contacted by telephone to consent for the treatment at the time of referral.

### Setting

The VVC provides service to a large expanse of Southwestern Ontario, with many patients residing over 200 km from the coordinating hospital. Travel to the central clinic for monitoring was not feasible. Therefore, a telephone and electronic-based monitoring system was established. The VVC team consisted of registered nurses and ID physicians. In Ontario, home IV medications, supplies and nursing care are publicly funded and made available through local health networks. Home care nurses from these networks administered vancomycin infusions in patients’ homes. The VVC provided the virtual monitoring and communicated the plan of care to both the patient and home care provider. Phlebotomy for monitoring occurred in outpatient laboratories local to the patient, as home care nurses in this region do not perform phlebotomy. All patients had an initial ID physician assessment, confirming that vancomycin was indicated and providing recommendations regarding initial vancomycin dose regimen, duration of therapy and trough level to be targeted. Patients had an initial assessment interview with nursing, either by phone or in person, at hospital discharge or within 24 h of referral. Nurses explained the process and rationale for monitoring clinical and laboratory data in detail and actively tracked results, with telephone and/or e-mail follow-up if any scheduled testing was missed. Patients were educated about the importance of the timed bloodwork in relation to infusion time, and laboratory appointments were scheduled accordingly. Rarely, trough results were found to be out of keeping with previously obtained baseline values. In those circumstances, a VVC nurse contacted the patient, determined if an error in timing had occurred, and requested a repeat test if appropriate. Patients remained under VVC care throughout the course of treatment. An ID physician provided consultation if dosing adjustments were required or if clinical concerns arose. A pharmacist was also available for consultation if needed. Follow-up of the condition necessitating vancomycin therapy was arranged with ID physicians as specified at their original consultation, or earlier if indicated during monitoring.

This study was approved by Western University’s Research Ethics Board. The data were anonymized prior to analysis. As this was a retrospective review of a quality improvement project for which the participants had provided consent to participate, additional informed consent to be included in the study was not required.

### Data collection method

Blood test monitoring included vancomycin trough levels and serum creatinine (Cr), performed twice weekly until vancomycin troughs were consistently within target, then weekly. Complete blood counts were performed weekly. VVC nurses actively monitored laboratory indices through the electronic health record linked with regional laboratories (the Ontario Laboratory Information System [OLIS]). Vancomycin troughs outside of target, other abnormal bloodwork results and new clinical developments were discussed by the nursing and ID clinician team, and adjustments to treatment were made as required. Nurses monitored online laboratory databases daily to review results and confirm that bloodwork was performed as scheduled. Nurses then sent telephone and/or e-mail messages to patients reminding them of scheduled bloodwork, following up on missed bloodwork within 24 h (including problem-solving and rescheduling), and notifying them of vancomycin dose adjustments. During patient encounters, any patient-reported symptoms or side effects were noted and documented in the electronic patient record and brought to physician attention as indicated. Nursing time was recorded per encounter in 15 min intervals, rounded up to the nearest 15 min.

### Outcome

The primary outcome was incidence of any clinically significant ADE or complication, determined through clinical assessment, laboratory findings, patient self-report and chart review. The secondary outcomes were readmission and mortality.

### Statistical analysis

All statistical analyses were performed using IBM SPSS Statistics version 26. Descriptive analyses were performed for demographic, clinical, microbiological and outcome data. χ^2^ and Fisher’s exact test were used to demonstrate the association between categorical variables. Paired *t*-test and Wilcoxon signed-rank test were used to compare quantitative variables (e.g. serum Cr level) before and after treatment. *P *<* *0.05 was considered statistically significant.

### Variables

We collected demographic information, history of using illicit drugs, microbiological diagnoses, clinical indication for vancomycin therapy, target serum vancomycin level, duration of treatment (i.e. number of days that parenteral vancomycin was administered), number and duration of patient-nurse encounters, concurrent antibiotic therapy (i.e. receiving at least one dose of another antimicrobial), vancomycin trough level (mg/L) and serum Cr. The indication for therapy was drawn from the assessment by the ID physician; where multiple clinical syndromes which would require IV therapy existed (e.g. infective endocarditis and septic arthritis) the case was categorized under the more severe syndrome (i.e. one determining the length of therapy or trough vancomycin level). The duration of follow-up was defined as the number of days between the day of referral and the day of treatment completion. Acute kidney injury (AKI) was defined as in previous consensus guidelines: Cr increase from baseline by ≥44 μmol/L (0.5 mg/dL) or to ≥150% of baseline, on ≥2 consecutive readings.^5^ To increase sensitivity, patients who did not have a second Cr measurement but whose single follow-up Cr met the numerical criteria were categorized as AKI. We further stratified renal dysfunction in stages based on previously published criteria (NB: these criteria, developed for inpatients, include urine output; our staging omitted this unavailable information and included only Cr results or the need for renal replacement therapy).^6^ Stage 2 AKI was defined as a rise in Cr 2.0- to 2.9-fold above baseline. Stage 3 AKI was defined as any of: a rise of ≥3.0-fold above baseline, an increase in Cr to ≥353.6 μmol/L (4 mg/dL) or initiation of renal replacement therapy.

Significant ADEs were defined as in previous studies: any event necessitating change in antimicrobial agent, early termination of medications, readmission, or *C. difficile* infection.^3^

Reporting of all aspects of this study adhered to the Strengthening Observational Studies in Epidemiology (STROBE) guidelines for observational cohort studies.^7^

## Results

**Figure 1.**
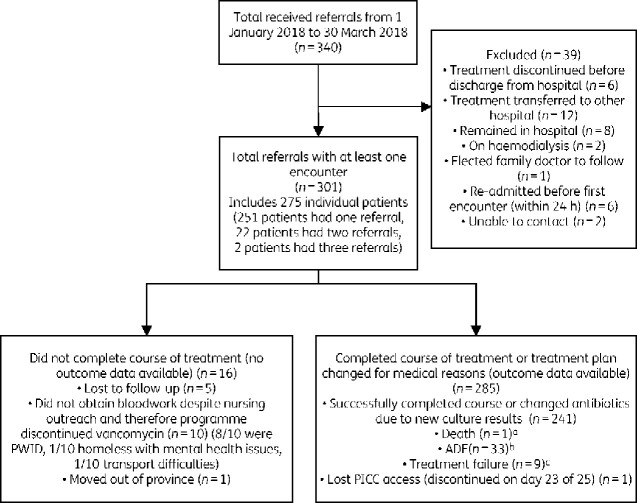
CONSORT diagram. ^a^Death due to metastatic malignancy and conversion to comfort care only; not related to treatment. ^b^See Table 2. ^c^Treatment failure: three involved IV line-associated bacteraemia (3/301, 1%), one patient non-adherence to therapy (1/301, 0.33%) and four worsening infection necessitating a new antibiotic regimen (4/301, 1.3%). One patient was re-admitted due to social reasons (homelessness and inability to receive home care reliably) (1/301, 0.33%). PICC, peripherally inserted central catheter.

Three hundred and forty referrals to the VVC were received during the study period. Of those, 301 had at least one encounter with a VVC nurse and were included in the analysis, capturing a total of 301 courses of OPAT with vancomycin in 275 patients (Figure 1).

The median/mean (range) numbers of nursing patient encounters (telephone or email) and laboratory test orders were 5/5.47 (1–19) and 3/3.89 (0–16), respectively. Median (IQR) duration of prescribed treatment with vancomycin was 42 (28–42) days while median (IQR) duration of therapy under the care of the VVC was 28 (14–42) days, with the remainder of days consisting of in-hospital vancomycin therapy. Nursing monitoring costs were $46.75 CAN/h ($35.42 USD) and totaled $63.93 CAN/patient ($48.43 USD) for a mean of 1 h 22 min monitoring per patient.

Table 1 shows demographic variables, indications for treatment, organisms isolated from culture media and concurrent antibiotic therapies. Bone and joint infections were the most common indication (43.5%). One hundred and forty-nine patients (49.5%) required concurrent antibiotic therapy. Cephalosporins were the most common concurrent antibiotic (17.9%). Sixty-eight of 301 treatment courses (22.6%) were based on empirical therapy.

**Table 1.** Characteristics of cohort

Variable	Frequency, *n *=* *301	%
Age, median (IQR), years	60 (45–68)	—
Female	118	39.2
PWID	21	7.0
Referring service		
orthopaedics	114	37.9
medicine	46	15.3
cellulitis clinic	30	10
neurosurgery	21	0.7
cardiovascular surgery	16	5.3
gastroenterology	12	4
cardiology	11	3.7
infectious diseases	10	3.3
otolaryngology	8	2.7
general surgery	6	2
plastic surgery	6	2
thoracic surgery	5	1.7
urology	5	1.7
nephrology	3	1
other	7	2.3
Indications for treatment		
infected joint (with or without hardware)	91	30.2
skin and soft tissue infection	44	14.6
osteomyelitis	40	13.3
bacteraemia	34	11.3
surgical site infection (non-joint related)	27	9
endocarditis	22	7.3
meningitis/epidural abscess	16	5.3
intra-abdominal infection	13	4.3
device-related infection (excluding bacteraemia)	8	2.7
pneumonia with *S. aureus*	2	0.7
other	4	1.3
Isolated microorganisms		
MRSA	102	33.9
* *coagulase-negative *Staphylococcus*	90	29.9
* Enterococcus* spp.	20	6.6
MSSA	6	2.0
* Cutibacterium acnes*	5	1.7
culture negative	68	22.6
other	10	3.3
Concurrent antimicrobial therapy		
none	152	50.5
cephalosporin	54	17.9
rifampicin	17	5.6
piperacillin/tazobactam	34	11.3
carbapenem	24	8.0
fluoroquinolone	6	2.0
trimethoprim/sulfamethoxazole	2	0.7
aminoglycosides	2	0.7
metronidazole	2	0.7
tetracycline	1	0.3
clindamycin	1	0.3
antivirals	2	0.7
oral vancomycin	1	0.3
daptomycin	1	0.3
Source of culture specimen		
tissue	65	21.6
blood	52	17.3
fluid	22	7.3
urine	1	0.3
wound	67	22.3
historic positive	20	6.6
culture negative	73	24.3
missing	1	0.3
Target level of vancomycin trough level		
10–15 mg/L	103	34.2
15–20 mg/L	125	41.5
10–20 mg/L	73	24.2

Overall, 285/301 patients (94.7%) completed the course of treatment. Seven of 285 (2.6%) required therapy modification due to isolation of new microorganisms from the site of infection. Thirty-three patients (11.0%, 95% CI 7.7%–15.1%) developed ADEs requiring discontinuation (Table 2). Fifteen of 33 (45.5%) ADE were due to renal toxicity (15/301 of total treatment courses, [5.0%, 95% CI 2.8%–8.1%]). Two of the 15 developed stage 2 AKI. No patients developed stage 3 AKI. Nine of 301 (3.0%) required readmission for treatment failure. Of the nine treatment failures, three involved IV line-associated bacteraemia (3/301, 1%), one patient non-adherence to therapy (1/301, 0.33%) and four worsening infection necessitating a new antibiotic regimen (4/301, 1.3%). One patient was re-admitted due to social reasons (homelessness and inability to receive home care reliably) (1/301, 0.33%). One patient died of complications of metastatic cancer after transition to comfort-focused care; death was not related to treatment of infection.

**Table 2.** Discontinuation of vancomycin due to adverse events (*n *=* *33)

Adverse events	Frequency, *n *=* *33
Renal changes	15
did not meet criteria for AKI	6
met criteria for AKI	5
possibly met criteria for AKI but second Cr not available	4
Other adverse events	18
skin rash	7
neutropenia	5
red man syndrome	1
nausea or vomiting	1
diarrhoea (*C. difficile* negative)	1
malaise, reduced appetite	1
tinnitus, headache	1
drug rash with eosinophilia and systemic symptoms	1

AKI was defined as in previous consensus guidelines: Cr increase from baseline by ≥44 μmol/L (0.5 mg/dL) or to ≥150% of baseline, on ≥2 consecutive readings.^5^

Table 3 shows serum Cr and vancomycin trough levels at baseline and at treatment completion. Median (IQR) baseline Cr was 71 (60–94) μmol/L (0.81 [0.68–1.1] mg/dL), which did not significantly change while patients continued treatment (Cr level at completion median [IQR]: 68 [56–87] μmol/L, *P *=* *0.68). Vancomycin trough levels did increase during this interval, with a significant difference between the first and the last vancomycin trough levels (median [IQR]: 10.2 [7.3–13.9] mg/L versus 14.4 [11.6–17.0], *P *<* *0.001); (mean difference: 3.76 mg/L, 95% CI 2.69–4.84).

**Table 3.** Serum Cr and vancomycin trough levels

Variable	Median (IQR)
Serum Cr before receiving vancomycin, μmol/L; [mg/dL]	71 (60–94); [0.80 (0.68–1.06)]
Initial serum Cr at VVC, μmol/L; [mg/dL]	68 (57–87); [0.77 (0.64–0.98)]
Last serum Cr at VVC, μmol/L; [mg/dL]	78 (64–96); [0.88 (0.72–1.08)]
Initial vancomycin trough level at VVC, mg/L	10.2 (7.3–13.9)
Pre-discharge vancomycin trough level, mg/L	14.4 (11.6–17)
Pre-discharge serum Cr level, μmol/L; [mg/dL]	68 (56–87); [0.77 (0.65–0.98)]

To evaluate the efficacy of VVC in preventing AKI when vancomycin trough levels were initially supratherapeutic, we performed a subgroup analysis of patients whose vancomycin trough level at the time of referral was >20 mg/L (*n *=* *20). In non-parametric analysis, the initial vancomycin trough level (median [IQR]: 24.3, [22.4–28.6] mg/L) significantly improved during follow-up and remained in the therapeutic range (median [IQR]: 14.90 [11.8–19.8], *P *=* *0.03). In this group of patients, serum Cr at the time of referral was not significantly different from the last measurement (median [IQR]: 100.00 [77.35–148.35] μmol/L versus 111.0 [81.0–144.5] μmol/L, *P *=* *0.36).

We also analysed patients whose initial Cr was >100 μmol/L (*n *=* *43). In this group, Cr at the time of referral was not significantly different from the last measurement (median [IQR]: 131 [107–167] μmol/L versus 131 [106–164.3] μmol/L, *P *=* *0.76).

There was no difference in incidence of AKI in patients who concurrently received piperacillin/tazobactam (2/34, 5.9%) versus patients who concurrently received a cephalosporin or carbapenem (4/78, 5.1%) (*P *=* *0.59).

## Discussion

To our knowledge this is the largest case series of OPAT vancomycin therapy and the first to report outcomes associated with a nursing-led active-monitoring programme using an integrated regional electronic database and telephone/e-mail follow-up. Our data demonstrate that vancomycin can be administered safely within an OPAT programme using our VVC approach. The incidence of vancomycin discontinuation due to renal adverse events was 15/301 patients (5.0%, 95% CI 2.8%–8.1%), while the incidence of discontinuation due to serious ADEs was 33/301 (11%, 95% CI 7.7%–15.1%). By comparison, Keller *et al.*,^8^ using common definitions, found that in their outpatient therapy cohort 11/89 patients (12.4%) developed AKI and 19/89 patients (21.3%) required vancomycin discontinuation due to serious adverse events.

There is a paucity of studies on the safety of vancomycin in OPAT programmes. Most vancomycin safety studies have been limited to inpatients, and these demonstrated a wide range in vancomycin-associated AKI incidence. A meta-analysis by van Hal *et al.* reported a prevalence of vancomycin-associated AKI ranging from 5%–43%.^9^ Higher troughs (>15 mg/L) were associated with increased odds of nephrotoxicity (OR 2.67; 95% CI 1.95–3.65). A meta-analysis of 13 randomized controlled studies reported a relative risk of AKI with vancomycin of 2.45 (95% CI 1.69–3.55), with an attributable risk of 59%.^10^ In critically ill patients, renal function often fails to fully recover after AKI, and even mild AKI can significantly decrease long-term survival; data on the impact of AKI in stable outpatients are lacking.^11^^,^^12^ Co-administration of piperacillin/tazobactam was associated with an increased risk of AKI in some studies^13^ though not in others.^14^ Our study did not find an increased risk of AKI in outpatients treated concurrently with piperacillin/tazobactam when compared with those treated concurrently with a cephalosporin or carbapenem, although our sample size of patients concurrently on β-lactams may have been inadequate to detect a difference.

An OPAT safety bundle has been proposed consisting of: patient selection, ID consultation, discharge planning, outpatient monitoring/tracking and OPAT programme review.^15^^,^^16^ Our approach included these processes, with the additions of nurse-directed active monitoring of laboratory results and telephone/e-mail contact with patients adding an additional level of safety. We attribute our greater safety results to active, anticipatory monitoring by nurses (looking for expected results, integrating reminders and following up on missing results) which led to follow-up with patients who otherwise would have defaulted from laboratory monitoring. This approach innovates on the most common OPAT structure, in which laboratory monitoring is performed by physicians; while standard systems send laboratory results to physician attention as they become available, there is no such standardization for flagging missing expected results.^17^ It also expands positively on other nurse-led programmes, e.g. Mansour *et al*.,^18^ in that our nurses reached out directly to patients, triggering needed monitoring and ensuring regular clinical contact and reassessment. In this way our VVC capitalized on the particular strengths of nursing involvement, affordably creating a reliable, pro-active system integrating direct patient care. This approach in concert with maintaining low trough vancomycin levels^4^ in these stable outpatients (median last trough 14.4) likely led to our improved patient safety outcomes.

Our success in reducing nephrotoxicity was not at the expense of adequate dosing, as only 4/301 (1.3%) developed worsening infection and need for a new antibiotic regimen. Furthermore, vancomycin dosing was appropriately adjusted based on bloodwork, such that patients with baseline elevated Cr or initially supratherapeutic vancomycin trough levels at hospital discharge were able to be safely continued on therapy. Our approach also allowed successful patient management in the home without hospital visits, a noteworthy benefit in the context of the present COVID-19 pandemic.

Clinical guidelines at the time of this study recommended monitoring of vancomycin trough concentrations.^5^^,^^19^ Revised dosing guidelines for vancomycin for treatment of serious MRSA infections have recently been published and recommend targeting an AUC of 400–600 mg·h/L using Bayesian dosing, rather than targeting trough levels.^20^ Reducing the incidence and complications of AKI provides strong motivation for the change to AUC monitoring^20^ along with reducing attributable mortality associated with ongoing bacteraemia via optimizing AUC. However, changes to an AUC target would be a particular challenge for OPAT programmes, which may lack dedicated pharmacy staff support for dosing and monitoring. The viability of trough only dosing using a Bayesian approach to identify the AUC needs more evaluation^20^ and OPAT programmes may be unable to obtain the needed peak and trough levels to optimize AUC calculations, given the timing constraints and current physical separation between phlebotomy and infusion. Therefore, AUC dosing would be particularly difficult when vancomycin therapy is initiated as an outpatient, as it was in 58 (19%) of our patients. Continuous vancomycin infusion as a strategy to ease some of these concerns in OPAT programmes is discussed in the new guidelines. Continuous vancomycin infusion in OPAT was suggested to be associated with a lower risk of nephrotoxicity in one study^21^ but not in others.^22^^,^^23^ However, even in the study which found a lower risk with continuous infusion, the nephrotoxicity rate noted (6/74, 6.8%) was not lower than that seen in our nurse-led VVC using intermittent infusion. At present, continuous infusion of vancomycin in OPAT programmes remains uncommon.^24^ Our data cannot be generalized to inpatients, who are often haemodynamically and biochemically unstable and may therefore require more advanced dosing algorithms; and indeed it is these patients who are directly addressed by the new AUC guidelines. However, our data demonstrate that very low incidence of both AKI and mortality can be achieved in stable outpatients receiving vancomycin in a VVC performing monitoring which targets trough levels. Further study of the optimal dosing strategy for vancomycin in the OPAT setting is warranted. 

Limited physician involvement made the programme feasible and relatively inexpensive, even within our large region and despite limited ID consultant availability. The integration of local electronic laboratory databases over a large geographic region, which allowed us to follow the large number of patients studied and capture extensive documentation regarding adverse drug reactions, contributed to the program’s success. However, this model may not be feasible in regions with less integrated data systems. Similarly, our model includes OPAT nursing availability every day of the week and daily access to on-call ID staff. This may not be available in other regions.

A limitation for our study was logistical, as in our region outpatients usually receive vancomycin in their homes via provincial home care programme, while home care nurses do not perform phlebotomy. While nurse-led teaching ensures that patients understand the importance of obtaining time-sensitive trough levels, geography can nonetheless make this bloodwork challenging for many patients, as they must have bloodwork done in a laboratory then travel home to receive their next dose. Integrated phlebotomy performed by home-care nurses would simplify our approach and warrants further study and funding.

Costs cited above include only that of nursing monitoring, excluding costs of laboratory testing (generally $8.40 CAD per laboratory visit), home care and IV equipment. These costs and, crucially, drug acquisition costs can vary widely between jurisdictions; however, they were not appreciably different from usual outpatient vancomycin care. Regardless, the VVC was clearly less expensive than ongoing inpatient vancomycin monitoring.

Social factors played a role in programme efficacy, as 8/10 patients who failed to obtain follow-up blood work and therefore required discontinuation of therapy were persons who inject drugs (PWID), and 1/10 was homeless. As 8/21 PWID (38%) required treatment interruption, the VVC may be less effective in this group. However, this may be surmountable, as recent studies have reported successful OPAT administration in homeless patients (including some PWID) using funded respite housing.^24–26^

A necessary limitation in our analysis was its restriction to patients referred to the VVC, thus excluding patients needing only brief courses of home IV vancomycin (≤5 days) not requiring monitoring. Such patients are generally at reduced risk for ADE, including AKI from vancomycin, thus reducing the impact of this limitation.

### Conclusions

A nurse-led VVC can safely actively monitor OPAT vancomycin therapy with a low rate of adverse events including AKI, a low incidence of treatment failure and low cost. Our key intervention, active anticipatory monitoring, ensuring minimal loss to follow-up, is broadly available and cost-effective. Assessment of other vancomycin dosing strategies within the OPAT environment to optimize safety and efficacy is warranted. Future research into the cost-effectiveness of programme delivery for specific patient populations would be desirable.
